# A descriptive study of potential participant preferences for the design of an incentivised weight loss programme for people with type 2 diabetes mellitus attending a public hospital in Lima, Peru

**DOI:** 10.12688/wellcomeopenres.14552.2

**Published:** 2018-09-27

**Authors:** Harold Akehurst, M. Amalia Pesantes, S. del Pilar Cornejo, Katty Manrique, Maria Lazo-Porras, Jill Portocarrero, Francisco Diez-Canseco, Antonio Bernabe-Ortiz, Antonio J. Trujillo, J. Jaime Miranda

**Affiliations:** 1 Great Western Hospitals NHS Trust, Swindon, UK; 2 CRONICAS Center of Excellence in Chronic Diseases, Universidad Peruana Cayetano Heredia, Lima, Peru; 3 Department of Endocrinology, Hospital Nacional Arzobispo Loayza, Lima, Peru; 4 CONEVID Unidad de Conocimiento y Evidencia, Universidad Peruana Cayetano Heredia, Lima, Peru; 5 Faculty of Epidemiology and Population Health, London School of Hygiene and Tropical Medicine, London, UK; 6 Department of International Health, Johns Hopkins Bloomberg School of Public Health, Baltimore, MD, USA; 7 School of Medicine, Universidad Peruana Cayetano Heredia, Lima, Peru

**Keywords:** Diabetes, Obesity, Motivation, Weight loss, Public health

## Abstract

**Background:** Weight loss is important for the control of type 2 diabetes mellitus but is difficult to achieve and sustain. Programmes employing financial incentives have been successful in areas such as smoking cessation. However, the optimum design for an incentivised programme for weight loss is undetermined, and may depend on social, cultural and demographic factors.

**Methods:** An original questionnaire was designed whose items addressed respondent personal and health characteristics, and preferences for a hypothetical incentivised weight loss programme. One hundred people with type 2 diabetes mellitus were recruited to complete the questionnaire from the endocrinology clinic of a public hospital in Lima, Peru. A descriptive analysis of responses was performed.

**Results:** Ninety-five percent of subjects who had previously attempted to lose weight had found this either 'difficult' or 'very difficult'. Eighty-five percent of subjects would participate in an incentivised weight loss programme. Median suggested incentive for 1 kg weight loss every 2 weeks over 9 months was PEN 100 (~USD $30). Cash was preferred by 70% as payment method. Only 56% of subjects would participate in a deposit-contract scheme, and the median suggested deposit amount was PEN 20 (~USD $6). Eighty percent of subjects would share the incentive with a helper, and family members were the most common choice of helper.

**Conclusions:** The challenge of achieving and sustaining weight loss is confirmed in this setting. Direct cash payments of PEN 100 were generally preferred, with substantial scope for involving a co-participant with whom the incentive could be shared. Employing direct financial incentives in future weight loss programmes appears to be widely acceptable among people with type 2 diabetes mellitus.

## Introduction

Weight control is critical for both prevention and treatment of type 2 diabetes mellitus (T2DM) ^1– 3^. Self-management programmes for people with T2DM commonly include the promotion of lifestyle changes, such as dietary modifications and increasing physical activity, to reduce weight ^4– 6^. However, sustained weight loss is a challenge to both patients and providers: failure to sustain weight loss in formal diet programmes varies between 21–54%, and many people fail repeatedly ^7– 9^.

A major challenge in any lifestyle intervention programme is the willingness to join, and sustain, participation. Better understanding of what motivates people to engage with such programmes is therefore fundamental to their design ^10^. Financial incentives have emerged as strategies which can initiate and sustain positive health behaviours during the incentive period and beyond. Sustained changes have been achieved through incentivization in the field of smoking cessation, but trials of financial incentives have previously failed to achieve sustained weight loss ^11– 14^.

Social and cultural factors influence participants’ engagement with weight control: for example, among adolescents, weight loss attempts were more frequent in Latinos than Whites or African Americans; desire to lose weight is more common in Latino females than males; and intention to lose weight is related to the number of social contacts trying to lose weight ^15– 17^. Successful completion of both short- and long-term weight loss programmes has been associated with age, ethnicity, family structure, educational level and employment ^18, 19^. Additionally, a recent study testing a behavioural weight loss intervention for Latinos in the United States concluded that companionship for physical activity appears to support weight loss ^20^. It is possible that the success of an incentivised weight loss intervention might be optimised by accounting for the social and cultural characteristics of its target population, and by incorporating beneficial social support by design.

Healthcare in Peru is funded publicly and privately: approximately 30% in the lowest socioeconomic stratum is covered by public health insurance (SIS); a further 25% are covered by social security (EsSalud) linked to their employment; 2% have private health insurance; and 38% have no health insurance. Separate military, police and other systems account for the remainder. Insulin, metformin and glyburide are available through SIS and EsSalud, while many further agents are available privately. Glucose testing strips are available for insulin-dependent diabetes through SIS and EsSalud, or through private insurance. There is no national strategy for diabetes care which integrates medical therapy with promotion of exercise or healthy diet ^21^.

The aim of this study was to investigate the optimal design for an incentivised weight loss programme which is planned for people with T2DM in Lima, Peru (to be funded by research grants). The objectives of the study were: to determine the acceptability of financial incentives for weight loss among type 2 diabetics in Lima, Peru; and to determine the optimal amount and delivery method for such an incentive.

## Methods

### Design and data collection

We performed a cross-sectional exploratory study using an original questionnaire. Interviews were conducted and data recorded by JP.

### Questionnaire development and design

The questionnaire was developed by the authors and not validated separately. It consisted of 82 items (see Supplementary Material for the instrument in original Spanish and translation) addressing socio-economic circumstances, health characteristics and preferences relating to a proposed incentivised weight loss programme. Items relating to the programme included a suggested incentive amount and identifying a threshold incentive amount.

Two methods were employed to identify threshold incentive amounts for participation in a weight loss reduction programme: direct questioning and fixed-increment questioning ( Supplementary Table 1 and Supplementary Table 2).

For the first method, a hypothetical situation was explained to the participant, which consisted of inviting them to participate in a 9-month programme whose purpose was to pay a monetary incentive only if they lost 1 kilogram every two weeks, and that we were interested in knowing the exact amount of money that would motivate them to lose that kilogram. For the second method, amounts of money from 0 PEN to 250 PEN in fixed increments of 50 PEN were specified and the participant was asked whether each of these amounts would motivate them to lose 1 kilogram over two weeks.

Participants were also asked about their willingness to participate in a hypothetical ‘deposit-contract’ programme in which they would be required to deposit a certain amount of money in a saving account and such amount would be doubled if they lost 1 kilogram over a two-week period, but would lose the deposited amount if they failed to reach the weight loss goal.

Finally, participants were asked if they would be willing to share the money won in a weight loss programme with a co-participant, defined as a relative or friend selected by the participant to support their efforts to lose weight, their preferred co-participant, and the proportion of the incentive that the participant would be willing to share with this co-participant.

### Participants

Patients were recruited by convenience sampling from the Hospital Nacional Arzobispo Loayza, a public tertiary hospital serving mostly low-income people from Lima, the capital city of Peru, whose endocrinology department provides over 2500 outpatient appointments annually to patients with T2DM ^22^.

Inclusion criteria were age ≥18 years and self-reported diagnosis of T2DM. Incapacity to provide written informed consent was the only exclusion criterion. As patients were attending an endocrinology clinic it was not considered necessary to verify their self-reported T2DM status independently, while the research team did not have access to participants’ medical records. Due to the exploratory nature of the study, only 100 subjects were invited to participate. Participants were recruited in the waiting room of the Endocrinology Department during April 2016.

### Data analysis

A descriptive analysis of questionnaire items was undertaken, employing 95% confidence intervals for selected items whose measurement was considered particularly important. For non-parametric continuous variables, a bootstrap confidence interval of the median was attempted. Hypothesis testing was not performed due to the large number of possible comparisons relative to the sample size and the consequently elevated risk of type 1 error. Statistical analysis was performed using R version 3.4.3 ^23^.

### Ethics

This study was approved by the Institutional Review Boards of the Universidad Peruana Cayetano Heredia (SIDISI 64789) and the Hospital Nacional Arzobispo Loayza (Expediente 04974-2015), in Lima, Peru. Written informed consent for participation was obtained from all subjects.

## Results

One hundred people with T2DM participated in the study. Two subjects did not respond to questions relating to incentives; the data were otherwise complete. Demographic and socioeconomic characteristics are presented in Table 1. Health-related responses are presented in Table 2. Measures previously taken to improve health are presented in Table 3.

**Table 1.** Demographic and socioeconomic characteristics of patients with type 2 diabetes included in the study.

Characteristic		Count (%) or Mean (Standard deviation)
Female sex		67 (67%)
Age		55 years (11.8)
Education	Primary completed	7 (7%)
	Secondary incomplete	4 (4%)
	Secondary completed	46 (46%)
	Further non-university incomplete	19 (19%)
	Further non-university completed	13 (13%)
	University incomplete	9 (9%)
	University completed	2 (2%)
Employed		55 (55%)
Household monthly income	< PEN 750 [< US $228]	3 (2%)
	PEN 751 – 1500 [US $228 – 456]	14 (14%)
	PEN 1501 – 2000 [US $456 – 608]	22 (22%)
	PEN 2001 – 2500 [US $608 – 760]	24 (24%)
	> PEN 2501 [> US $760]	11 (11%)
	Refused to answer	26 (26%)
Health insurance	None	34 (34%)
	Sistema Integral de Salud (most basic insurance)	64 (64%)
	Essalud (state-provided insurance for the employed)	2 (2%)
Self-rated economic status	Very bad	1 (1%)
	Bad	19 (19%)
	Fair	47 (47%)
	Good	33 (33%)

**Table 2.** Health characteristics of patients with type 2 diabetes included in the study.

Variable		Count (%) or Mean (standard deviation)
Self-rated health status	Very bad	11 (11%)
	Bad	52 (52%)
	Fair	37 (37%)
Time since diagnosis of diabetes		6.9 years (5 years)
Most recent blood glucose measurement (self-reported)	Reported (n = 94)	151 mg/dL (49 mg/dL)
	Did not know	6 (6%)
Most recent HbA1c measurement (self- reported)	Reported (n = 59)	8.9% (1.6%)
	Did not know	41 (41%)
Current medical treatment for diabetes	Any	95 (95%)
	Insulin	10 (10%)
	Metformin	71 (71%)
	Glibenclamide	32 (32%)
	Glimepiride	1 (1%)
	Weight loss tablets	1 (1%)
Monthly expenditure on medical treatment for diabetes	PEN [US $]	63 (44) 19 (14)

**Table 3.** Measures previously taken to control health of patients with type 2 diabetes included in the study.

Health control measures attempted since diagnosis of diabetes		Count (%) or Mean (standard deviation)
Regular exercise		53 (53%)
Difficulty of attempt to regularly exercise	Very easy	1 (2)%
	Easy	21 (40%)
	Difficult	21 (23%)
	Very difficult	19 (40%)
Reduction of sugar intake		75 (75%)
Difficulty of attempt to reduce sugar intake	Very easy	1 (1%)
	Easy	32 (43%)
	Difficult	32 (43%
	Very difficult	9 (12%)
	Did not answer	1 (1%)
Quit alcohol		31 (31%)
Difficulty of attempt to quit alcohol	Very easy	1 (3%)
	Easy	19 (61%)
	Difficult	9 (29%)
	Very difficult	2 (6%)
Reduce fat intake		77 (77%)
Difficulty of attempt to reduce fat intake	Easy	27 (35%)
	Difficult	38 (49%)
	Very difficult	12 (16%)
Increase vegetable intake		57 (57%)
Difficulty of attempt to increase vegetable intake	Very easy	17 (30%)
	Easy	32 (56%)
	Difficult	8 (14%)
Weight loss		42 (42%)
Difficulty of attempt to lose weight	Easy	2 (5%)
	Difficult	23 (55%)
	Very difficult	17 (40%)
Methods for health maintenance or improvement for people with diabetes (all participants asked to name three)	Alternative medication	1 (1%)
	Attend appointments	4 (4%)
	Avoid appointments	1 (1%)
	Exercise	72 (72%)
	Foot care	6 (6%)
	Glycaemic control	13 (13%)
	Healthy diet	38 (38%)
	Intake control	17 (17%)
	Medications	38 (38%)
	Obey doctors	2 (2%)
	Reduce alcohol	1 (1%)
	Reduce carbohydrate	36 (36%)
	Reduce fat	19 (19%)
	Reduce protein	1 (1%)
	Relaxation	3 (3%)

**Figure 1.**
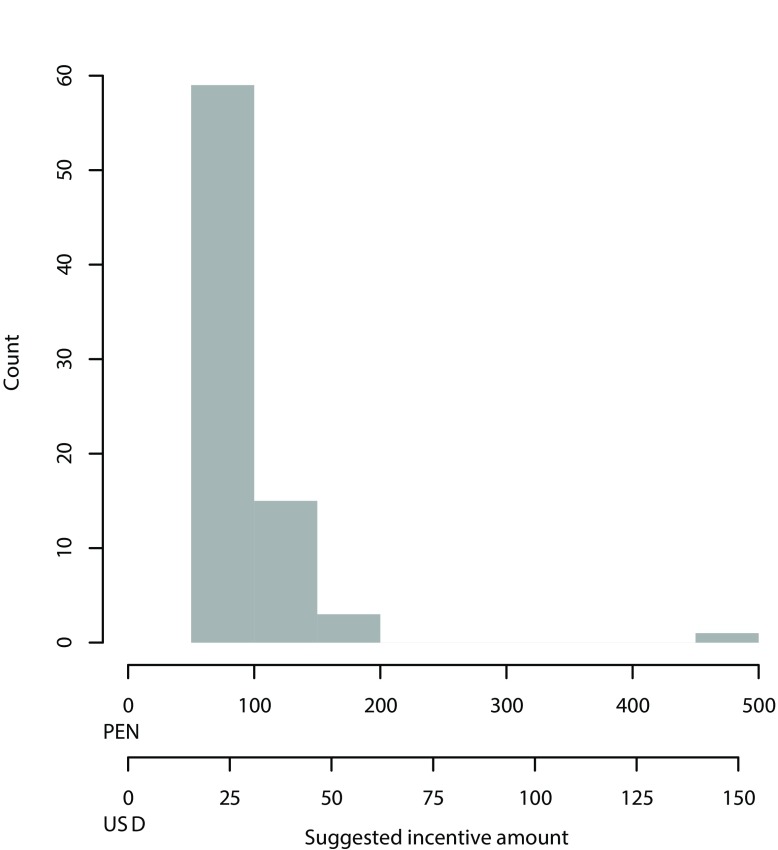
Suggested simple incentive amounts.

Ninety-eight subjects (98%) responded to questions about financial incentives. Ninety-two subjects (94%; 95% CI 87 – 97%) responded that they would participate in an unincentivised weight loss programme. Eighty-three (85%; 95% CI 76 – 91%) would participate in a 9-month incentivised weight loss programme. Reasons given for not participating included: insufficient time to attend biweekly follow-up visits; because they thought it would be difficult to avoid “antojitos” (cravings) for 9 months; or because the participant did not think they needed to lose weight. Seventy-eight subjects (78%) answered the question "how much money would motivate you to lose 1 kg every 2 weeks?". Responses were positively skewed with median PEN 100 (≈ USD $30) and range PEN 50 to 500 (≈ USD $15 to 150) ( Figure 1). Bootstrap confidence intervals could not be constructed because all resampled medians = PEN 100 (10,000 simulations).

Subjects were then asked whether they would participate in an incentivised weight loss programme with incentive amounts from PEN 50 to 250 in PEN 50 increments. Six subjects (6%) would not participate for any amount, while 91 (93%) would participate for all amounts. One subject changed from a positive to negative response at the PEN 200 threshold.

Asked about their preferred method of payment, 69 subjects preferred (70%) cash, 24 (25%) deposit into a bank account, 3 (3%) as vouchers and the remainder not responding.

**Figure 2.**
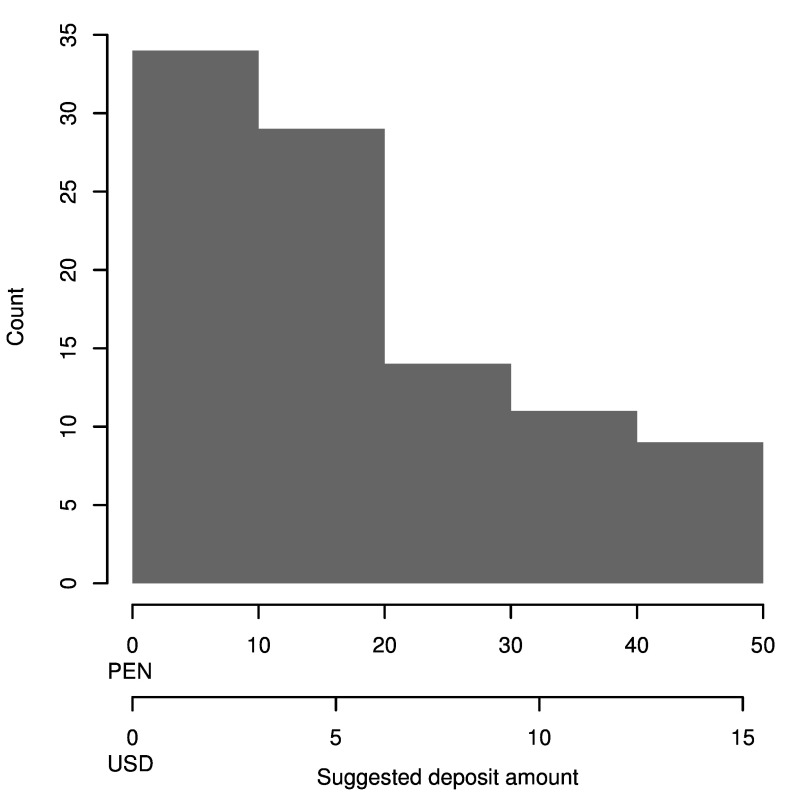
Suggested deposit amounts.

Fifty-five subjects (56%; 95% CI 46 – 66%) would participate in a deposit-contract scheme whereby their deposit would be doubled if they succeeded but lost if their failed to lose weight. Ninety-seven (97%) subjects answered a question on preferred deposit amount. Preferred deposit amount was positively skewed with median PEN 20 (≈ USD $6) and range PEN 0 to 50 (≈ USD $0 to 15) ( Figure 2). Again, equality of all resampled median precluded construction of bootstrap confidence intervals.

Subjects were then asked whether they would participate in a deposit-contract scheme with deposit amount in increments between PEN 25 – 250. Forty-three subjects would participate with any deposit amount (43%); 32 would not participate with any deposit amount (32%); and 22 identified a threshold deposit amount for participation (22%). Among subjects who identified a threshold deposit amount above which they would not participate, the maximum acceptable amount was positively skewed with median PEN 25 (range PEN 25 to 100).

Regardless of their answers to the previous questions, subjects were also asked for their views of participating in such a program. Out of the 73 who responded, 14 (19%) considered that it was not good to receive money for taking care of their own health, with one saying that this would be “like selling yourself”, since people should lose weight for their own sake and not for money. Sixteen (22%) said it was a good idea and were even excited at the prospect of participating in the program. Six (8%) found it amusing that such a program was even possible, and five (7%) were concerned that such a program will achieve only short-term results that would not be sustained after the program ended. Other answers revolved about the doubts they had about the program, or they did not understand the idea behind receiving money, that it was a good idea for “poor” people but not for everybody or that it might not work since not all diabetics needed to lose weight.

Subjects were asked who they would choose to help them to lose weight. Five (5%) chose a friend; 42 chose a partner (42%); 23 chose a child (23%); 1 chose a neighbour (1%); 4 chose a sibling (4%); and 12 would not choose a helper (12%). Eighty subjects would share the incentive with a helper (80%). Eight (10%) of these would share less than half, 71 (89%) half exactly, and 1 (1%) more than half of the incentive.

## Discussion

This pilot study aimed to characterise people with T2DM attending a public hospital in Lima, Peru, and their preferred amount and delivery method for a financial incentive to be used in a future incentivised weight loss programme.

The proportion of participants who would participate in an incentivised weight loss programme was high (85%) but the proportion who would participate in an unincentivised programme was even higher (94%). A similar pattern was observed in a mixed-methods study of acceptability of incentives for a weight loss maintenance programme, in which 93.9% supported the programme generally but only 77% supported cash incentives ^24^. The finding may indicate that in our sample weight loss as a goal was a more powerful motivator than the financial incentive. However, this does not negate the potential utility of incentives, which might contribute to participant retention and sustained weight loss achievement in addition to recruitment. It is possible another group exists, but was not accessed in this study, who do not wish to lose weight but could be motivated by financial incentives to do so. Such a group might be the most appropriate target for an incentivised weight loss intervention, but its access could constitute a significant challenge.

That fewer respondents would participate in an incentivised than in an unincentivised programme may also be due to unacceptability of financial incentives in this population for moral reasons: 14 thought it wrong to accept money in exchange for taking care of your own health, with one describing this as “selling yourself”. Similar concerns were expressed in focus groups in a recent study, in which discussion of financial incentives conveyed “distrust and indignation”, where the idea was reiterated that improved health should be sufficiently motivating for weight loss ^24^. These comments may represent a significant cultural attitude towards financial incentives for health which could constitute a barrier to their success, and which deserve further attention. However, such reservations may not necessarily preclude participation: 85% of subjects nonetheless indicated that they would participate in an incentivised intervention, and a recent systematic review found that participation may actually be increased by financial incentives for weight loss ^14^.

Median suggested incentive amount was PEN 100. Based on a national disposable income of USD $175.7bn ^25^ and population of 30,565,431 in 2013 ^26^, a maximum reward of PEN 100 every 2 weeks for 9 months would represent 10% of personal disposable income (PDI). Previous interventions have employed a broad range of incentive sizes (from 0.2% to 10.2% of PDI ^13^), and experimental evidence suggests that insufficient incentives may paradoxically produce less motivation than no incentive at all ^27^. The suggested amount therefore appears adequate and appropriate for an intervention in this setting.

The fixed-increment questioning method to identify a suitable incentive amount (asking whether the participant would accept amounts of increasing PEN 50 increments) was not successful. Sixty-two percent of participants in a previous study felt that financial incentives undermined individual responsibility for health ^28^, and participants may have been reluctant to engage with these questions to avoid weighing a moral position against financial advantage.

Fewer respondents would participate in a deposit-contract scheme, which concurs with previous findings ^24^. Because such schemes weigh a certain short-term price against a possible long-term advantage, they fail to take advantage of the established health economic principle that individuals overvalue present relative to future costs ^29^. In contrast, an approach described as *asymmetric paternalism*, which aims to assist individuals with health-improving behaviours without limiting freedom ^30^, might produce in an intervention in which individuals commit to future behaviours without present costs, such as receiving up-front an incentive which would be returned or doubled depending on achievement of a future weight goal. Cash or bank transfer were generally preferred over vouchers. This is in accordance with the finding that rewards are more motivating when separated from larger payments, such as household shopping (in the case of vouchers) and insurance premiums (in the case of discounts) ^31^.

Our findings show that most participants had found it challenging to adopt health-improving behaviours. In particular, 42% of participants had previously attempted to lose weight but 95% found this “difficult” or “very difficult”, suggesting that people with failed previous weight loss attempts will constitute a substantial subgroup of this population. The question of what makes behavioural change difficult has been addressed by Kelly & Barker, who note the mistakes which policy-makers commonly make in understanding the drivers of behaviour ^32^. One of these mistakes is the economic utility theory which presumes that individuals make rational choices to maximise gain and minimise loss. The theory behind the use of financial incentives is essentially an extension of this. However, health behaviours are frequently automatic responses to social and environmental cues, not subject to particular conscious reflection, and often in spite of adequate understanding of health implications ^33– 35^. These findings inform interventions that target ‘choice architecture’, comprising the “interaction between individual human agency and both the immediate and broader environment that make up the social structure” ^36^. Financial incentives are much more likely to achieve persisting behavioural change in synergy with such interventions.

Asked about who they would choose to help them to lose weight, most selected a family member. In prospective studies, family support was associated with reduced HbA1c in males, but increased HbA1c in females. Informal support seeking is often different in males and females. Females seek and receive more support from friends and extended family, while males often seek and receive more support from their spouse ^37^. Other studies found that seeing friends more frequently, having a well-functioning social network and a sense of good social support from the social network was associated with higher patient activation levels, less diabetes-related emotional distress and more health-promoting self-management behaviours among patients with T2DM. When providers felt more emotionally engaged, their support exerted a large, positive effect on their well-being, as well as on recipients’ well-being ^38, 39^. These findings imply that the incorporation of social support into an intervention may be crucial for its success, but also that its precise form may need to adapt to the sex (and potentially other characteristics) of the participant.

Five participants additionally raised concerns over the sustainability of weight loss in such a programme, and indeed this obstacle remains to be overcome: thus far, incentivised weight loss programmes have failed to achieve sustained weight loss beyond the incentivised period ^13, 14^. The Stages of Change model proposes pre-contemplation, contemplation, preparation, action and maintenance stages to behavioural change ^40^. To participate in the hypothetical 9-month intervention proposed to participants, they would necessarily have reached the ‘preparation’ stage but at the conclusion of the intervention might have spent only a maximum of 3 months in ‘maintenance’, presuming no relapses. Sustained weight loss would therefore not be incentivised for a long period in such an intervention. Indefinite incentive payment is unlikely to appeal to insurers or healthcare providers, nor is it likely to be evaluated due to its likely limited appeal to research funding bodies. Incentives may ultimately prove most valuable in initiating and achieving short to medium term change but will need to be integrated into a multimodal approach for treating T2DM and obesity more generally, including psychological, medical and potentially surgical methods (although these are unlikely to be available to this demographic for some time yet).

### Limitations

The sampling approach employed may have exposed the study to participation bias. Most participants were female, middle-aged, and had at least completed secondary education. Although most rated their economic status as at least ‘fair’, almost all had either the most basic or no health insurance at all. Although the prevalence of T2DM is greater in males than females worldwide ^41^, the higher proportion in our study may be explained by the fact that females are more likely than males to engage with healthcare seeking behaviours and respond to questionnaires ^42, 43^. The study setting in a Peruvian public hospital is likely to have determined participants’ socioeconomic profile, which should not be interpreted as representative of people with diabetes in Peru more generally. However, the prevalence of T2DM is inversely proportional to socioeconomic status ^44, 45^, and therefore the majority of people with T2DM in Peru will fall into the low-income group surveyed in this pilot and targeted by our planned intervention. Higher-educated subjects have previously been found to make more attempts to lose weight ^46^, which may imply a greater need for intervention in this low-income group.

Anthropomorphic and laboratory data relating to participants’ weight and diabetic control were not recorded and it was therefore not possible to examine whether responses were influenced by these. It is also unknown what proportion of participants were overweight or obese. The possibility exists that although participants reported that had T2DM and were attending an endocrinology clinic they may not have had T2DM, as this was not verified by laboratory testing because the authors did not have access to participants’ medical records.

The questionnaire used was original and not previously validated. Important parameters for an incentivised weight loss programme were not explored in our questionnaire. A ‘lottery’ form for payments, in which successful weight loss would allow entry into a regular lottery for a larger payment (and which is anticipated to be more motivating than direct payments because people tend to over-value small odds of large rewards ^31, 47^) was not proposed to participants. Participants were also not asked about their preferred frequency of payment. Higher-frequency payment have been shown to be more effective in the drug-abstinence setting ^48^, and the finding that experimental subjects prefer to segregate than to integrate gains has been used to support the argument for direct rewards over insurance premium adjustment ^31, 49^. These factors are important for the planning of any intervention and the preferences of potential participants should be the subject of future investigation.

Although the difficulty which participants had experienced in adopting health control behaviours was quantified, participants were not asked why each behaviour was difficult. This information could be usefully obtained through qualitative research, and might point to other potential targets for intervention, such as psychological, environmental and social factors.

Although multivariate associations could not be investigated due to insufficiency of sample size and sampling design, the study was not designed to investigate these, but rather to develop an improved understanding of the potential use of incentives in this setting.

## Conclusion

The use of direct financial incentives in a future weight loss programme for people with T2DM in Lima, Peru was acceptable to the majority of participants in this study, although some expressed reservations regarding the morality and sustainability of such a programme.

## Data availability

Original and translated data files are available on Open Science Framework: http://doi.org/10.17605/OSF.IO/8NQVW ^49^

Data are available under the terms of the Creative Commons Zero "No rights reserved" data waiver (CC0 1.0 Public domain dedication).

## Supplementary material

Supplementary Table 1: Questions asked to identify the maximum amount participants would accept as a financial incentive for weight loss [English translation].

Supplementary Table 2: Questions asked to identify the maximum amount participants would be willing to invest, in order to double their money upon meeting the weight loss target [English translation].

Supplementary File 1: Main questionnaire [English translation].

Supplementary File 2. Main questionnaire [Spanish].
