# Supplementary material for: A descriptive study of potential participant preferences for the design of an incentivised weight loss programme for people with type 2 diabetes mellitus attending a public hospital in Lima, Peru
Source: Wellcome Open Res. 2018 Sep 27;3:53. Originally published 2018 May 3. [Version 2] doi: 10.12688/wellcomeopenres.14552.2 (PMC6348435; doi:10.12688/wellcomeopenres.14552.2)
Supplement: Supplementary file 1 [file wellcomeopenres-3-16187-s0000.tgz › 15516ae6-76ed-428b-bfa1-37b5f2034549.docx]

### Supplementary Table 1: Questions asked to identify the maximum amount participants would accept as a financial incentive for weight loss [English translation].

| ¿Y aceptaría el reto de bajar 1 kilo en dos semanas si no le pago nada?  [Would you accept the challenge of losing 1 kg in two weeks if you were paid nothing?] |
| --- |
|  |
| ¿Y aceptaría el reto de bajar 1 kilo en dos semanas si le pago 50 soles?  [Would you accept the challenge of losing 1 kg in two weeks if you were paid PEN 50?] |
|  |
| ¿Y aceptaría el reto de bajar 1 kilo en dos semanas si le pago 100 soles?  [Would you accept the challenge of losing 1 kg in two weeks if you were paid PEN 100?] |
|  |
| ¿Y aceptaría el reto de bajar 1 kilo en dos semanas si le pago 150 soles?  [Would you accept the challenge of losing 1 kg in two weeks if you were paid PEN 150?] |
|  |
| ¿Y aceptaría el reto de bajar 1 kilo en dos semanas si le pago 200 soles?  [Would you accept the challenge of losing 1 kg in two weeks if you were paid PEN 200?] |
|  |
| ¿Y aceptaría el reto de bajar 1 kilo en dos semanas si le pago 250 soles?  [Would you accept the challenge of losing 1 kg in two weeks if you were paid PEN 250?] |
|  |
