# Supplementary material for: A descriptive study of potential participant preferences for the design of an incentivised weight loss programme for people with type 2 diabetes mellitus attending a public hospital in Lima, Peru
Source: Wellcome Open Res. 2018 Sep 27;3:53. Originally published 2018 May 3. [Version 2] doi: 10.12688/wellcomeopenres.14552.2 (PMC6348435; doi:10.12688/wellcomeopenres.14552.2)
Supplement: Supplementary file 2 [file wellcomeopenres-3-16187-s0001.tgz › 7ba43451-01b4-412b-94bd-e6dd6b2174ea.docx]

### Supplementary Table 2: Questions asked to identify the maximum amount participants would be willing to invest, in order to double their money upon meeting the weight loss target [English translation].

| ¿Pondría 25 soles de su bolsillo para ganar 50 soles si baja un kilo en dos semanas?  [Would you deposit PEN 25 of your own money to win PEN 50 if you lost 1 kg in two weeks? |
| --- |
|  |
| ¿Pondría 50 soles de su bolsillo para ganar 100 soles si baja un kilo en dos semanas?  [Would you deposit PEN 50 of your own money to win PEN 100 if you lost 1 kg in two weeks? |
|  |
| ¿Pondría 75 soles de su bolsillo para ganar 150 soles si baja un kilo en dos semanas?  [Would you deposit PEN 75 of your own money to win PEN 150 if you lost 1 kg in two weeks? |
|  |
| ¿Pondría 100 soles de su bolsillo para ganar 200 soles si baja un kilo en dos semanas?  [Would you deposit PEN 100 of your own money to win PEN 200 if you lost 1 kg in two weeks? |
|  |
| ¿Pondría 150 soles de su bolsillo para ganar 300 soles si baja un kilo en dos semanas?  [Would you deposit PEN 150 of your own money to win PEN 300 if you lost 1 kg in two weeks? |
|  |
| ¿Pondría 200 soles de su bolsillo para ganar 400 soles si baja un kilo en dos semanas?  [Would you deposit PEN 200 of your own money to win PEN 400 if you lost 1 kg in two weeks? |
|  |
| ¿Pondría 250 soles de su bolsillo para ganar 500 soles si baja un kilo en dos semanas?  [Would you deposit PEN 250 of your own money to win PEN 500 if you lost 1 kg in two weeks? |
