# Supplementary material for: A descriptive study of potential participant preferences for the design of an incentivised weight loss programme for people with type 2 diabetes mellitus attending a public hospital in Lima, Peru
Source: Wellcome Open Res. 2018 Sep 27;3:53. Originally published 2018 May 3. [Version 2] doi: 10.12688/wellcomeopenres.14552.2 (PMC6348435; doi:10.12688/wellcomeopenres.14552.2)
Supplement: Supplementary file 3 [file wellcomeopenres-3-16187-s0002.tgz › bf433b87-1570-4437-911f-992582b4d244_supp_file_1._Questionnaire_(English_translation).docx]

**CRÓNICAS**

**Centre of excellence in chronic diseases**

**Formative study part 1**

**Financial incentives for people with diabetes**

Please introduce yourself before beginning:

*“Good morning/afternoon, my name is … I am a member of healthcare staff from the Centre of Excellence in Chronic Diseases at the Peruana Cayetano Heredia University. We are carrying out a research study into the use of rewards to encourage people with diabetes to take better control over their disease.*

*You have been contacted because you have type 2 diabetes and because you receive treatment in this healthcare institution.*

*I would like to ask you some general questions about yourself. Beforehand I will explain the reasons for the study and I will ask you if you are interested in participating. Then I will give you an information sheet about the study and you will be able to decide whether you wish to participate.*

| **Section 1: Data about the interview and participant** |
| --- |

| **Module: interview data** | | **Responses** | | | | | |
| --- | --- | --- | --- | --- | --- | --- | --- |
| **1** | Interviewer code |  | 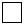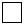 | | | | |
| **2** | Date (dd-mmm-20yy) |  | **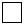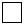** | **-** | **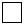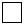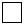** | **-** | **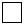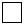** |

| **Module: demographic information** | | **Responses** | | | | | |
| --- | --- | --- | --- | --- | --- | --- | --- |
| **3** | Sex (record according to appearance) | 1 | Male |  |  |  |  |
|  |  | 2 | Female |  |  |  |  |
| **4** | Date of birth |  | **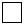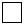** | **-** | **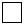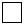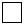** | **-** | **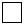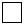** |
| **5** | Age in years | **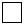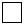** Años | | | | | |

| **Module: consent** | | **Responses** | |
| --- | --- | --- | --- |
| **6** | Has read consent | 1 | Yes |
|  |  | 2 | No **🡪 Read consent** |
| **7** | Consent of participant has been obtained | 1 | Yes |
|  |  | 2 | No **🡪 End the interview** |

***The information contained in this section must be stored separately from the questionnaire as it contains confidential information.***

| **Section 2: Demographic and socioeconomic data** | | | |
| --- | --- | --- | --- |
| **Module: demographic information** | | **Response** | |
| **8** | What is your civil status? | 1 | Single |
|  |  | 2 | Married |
|  |  | 3 | Cohabiting |
|  |  | 4 | Separated |
|  |  | 5 | Divorced |
|  |  | 6 | Widowed |
|  |  | 99 | Refuse to respond |
| **9** | What health insurance do you have?  ***(Read the options)*** | 1 | YESS |
|  |  | 2 | Essalud |
|  |  | 3 | None |
|  |  | 4 | Other: |
| **10** | What level of education have you attained?  ***(Choose only one option)*** | 1 | None |
|  |  | 2 | Pre-school |
|  |  | 3 | Primary, incomplete |
|  |  | 4 | Primary, completed |
|  |  | 5 | Secondary, incomplete |
|  |  | 6 | Secondary, complete |
|  |  | 7 | Further, incomplete |
|  |  | 8 | Further, complete |
|  |  | 9 | Higher, incomplete |
|  |  | 10 | Higher, complete |
|  |  | 88 | Doesn’t know/remember |
|  |  | 99 | Refuse to respond |
| **11** | Do you have a job/employment, either fixed or casual, for which you receive an income or salary? | 1 | Yes |
|  |  | 2 | No |
| **12** | What is your occupation or principle activity?  ***(Choose only one option)*** | 1 | Government employee |
|  |  | 2 | Private sector employee |
|  |  | 3 | Self-employed |
|  |  | 4 | Labourer |
|  |  | 5 | Farm-worker |
|  |  | 6 | Housewife |
|  |  | 7 | Domestic |
|  |  | 8 | Other: __________________________ |
|  |  | 99 | Refuse to respond |
| **13** | Besides your salary, do you have any other source of income?  ***(Read the options, choose all which apply)*** | 1 | Rent of property |
|  |  | 2 | Rent of equipment |
|  |  | 3 | Rent of land |
|  |  | 4 | Pension |
|  |  | 5 | Pension 65 (a type of pension) |
|  |  | 6 | Remittances from family members |
|  |  | 7 | Benefit for food, separation, widowhood, disability |
|  |  | 8 | Other: |
|  |  | 9 | None |
|  |  | 99 | Refuse to respond |
| **14** | Is your income used for personal expenses, family expenses, or both? | 1 | Personal expenses |
|  |  | 2 | Family expenses |
|  |  | 3 | Both |
|  |  | 4 | I have no income |
| **15** | Including yourself, how many people live in your home? Do not include domestic workers. | **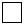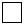** people | |
| **16** | Including yourself, how many people who live in your home have an income? | **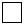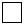** people have an income | |
| **17** | Add the incomes of all of the people who live in your home: yourself, your partner, your children, your parents etc. Include all sources of income: salaries, pensions, family support, rent etc. What is the monthly income of your household?  ***(Read the options. Choose only one)*** | 1 | < = 750 soles |
|  |  | 2 | Between 751 and 1500 soles |
|  |  | 3 | Between 1501 and 2000 soles |
|  |  | 4 | Between 2001 and 2500 soles |
|  |  | 5 | > 2501 soles |
|  |  | 88 | Doesn’t know/remember |
|  |  | 99 | Refuse to respond |
| **18** | How would you say is the economic situation of your household currently?  ***(Read the options. Choose only one)*** | 1 | Very Good |
|  |  | 2 | Good |
|  |  | 3 | Regular |
|  |  | 4 | Bad |
|  |  | 5 | Very bad |

| **Section 3: Health** |
| --- |

| **Module: health** | | **Response** | | |
| --- | --- | --- | --- | --- |
| **19** | How would you say that your health is, today?  ***(Read the options. Choose only one)*** | 0 | Very bad | |
|  |  | 1 | Bad | |
|  |  | 2 | Regular | |
|  |  | 3 | Good | |
|  |  | 4 | Very good | |
|  |  | 98 | Refuse to respond | |
| **20** | Do you know your approximate current weight? | 1 | Yes | |
|  |  | 2 | No **🡪 Proceed to question 22** | |
| **21** | Approximately how much do you weigh? | 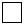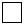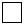**.**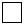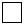 [Kg] | | |
| **22** | Would you like to …?  ***(Read the options. Choose only one)*** | 1 | Lose weight | |
|  |  | 2 | Maintain your current weight | |
|  |  | 3 | Increase your weight | |
|  |  | 98 | Doesn’t know/respond | |
| **23** | Do you know your approximate height? | 1 | Yes | |
|  |  | 2 | No **🡪 Proceed to question 25** | |
| **24** | Approximately what is your height | 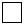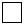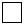 [Cm] | | |
| **25** | How long ago were you diagnosed with diabetes mellitus? | 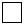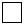 years ago, or | | 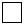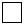 months ago |
| **26** | In the last 7 days, have your glucose levels been measured? | 1 | Yes | |
|  |  | 2 | No **🡪 Proceed to question 28** | |
|  |  | 98 | Doesn’t know/respond **🡪 Proceed to question 28** | |
| **27** | What was your glucose measurement? | 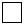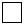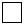(mg /dl) | | |
|  |  | 98 | No recuerda | |
| **28** | In the last 3 months, has your HbA1c been measured? | 1 | Yes | |
|  |  | 2 | No**🡪 Proceed to question 30** | |
|  |  | 98 | Doesn’t know/respond **🡪 Proceed to question 30** | |
| **29** | What was your HbA1c measurement? | 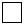 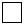. 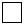 % | | |
|  |  | 98 | Doesn’t remember | |
| **30** | Currently, have you been prescribed any medication for the treatment of your diabetes? | 1 | Yes | |
|  |  | 2 | No **🡪 Proceed to the next module** | |
| **31** | Are you taking one of the following medications>  **(Read the options. Choose all which apply)** | 1 | Metformin | |
|  |  | 2 | Glibenclamide | |
|  |  | 3 | Insulin | |
|  |  | 4 | Other medication: | |
|  |  | 5 | Weight loss tablets | |
|  |  | 98 | Not taking medications | |
| **32** | Are you using these medications as prescribed? | 1 | Yes | |
|  |  | 2 | No | |
|  |  | 3 | Sometimes | |
| **33** | On average, how much do you spend monthly to buy your diabetic medication? | 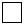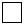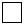Soles | | |
| **Module: knowledge about diabetes** | | **Response** | | |
| **35** | Are you able to tell me three ways in which a patient with diabetes can control their disease or improve their health? | A |  | |
|  |  | B |  | |
|  |  | C |  | |
| **36** | Do you practice any of these? | 1 | Yes | |
|  |  | 2 | No**🡪 Proceed to question 38** | |
| **37** | Which do your practice?  **(Choose all which apply)** | 1 | A | |
|  |  | 2 | B | |
|  |  | 3 | C | |
|  |  | 4 | All | |
| **38** | Have you ever received education about diabetes? | 1 | Yes | |
|  |  | 2 | No **🡪 Proceed to question 42** | |
| **39** | Where |  | | |
| **40** | How long did it last? (complete only one) | 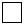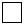 Days | | |
|  |  | 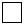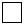Weeks | | |
|  |  | 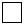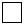Months | | |
|  |  | 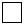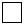 Years | | |
| **41** | Who taught you? |  | | |
| **42** | Since you were diagnosed with diabetes, have you ever tried to do regular exercise or physical activity> | 1 | Yes | |
|  |  | 2 | No **🡪 Proceed to question 44** | |
| **43** | How difficult was it for you?  ***(Read the options)*** | 1 | Very easy | |
|  |  | 2 | Easy | |
|  |  | 3 | Difficult | |
|  |  | 4 | Very difficult | |
| **44** | Since you were diagnosed with diabetes, have you ever tried to consume less sugar or sweets? | 1 | Yes | |
|  |  | 2 | No **🡪 Proceed to question 46** | |
| **45** | How difficult was it for you?  ***(Read the options)*** | 1 | Very easy | |
|  |  | 2 | Easy | |
|  |  | 3 | Difficult | |
|  |  | 4 | Very difficult | |
| **46** | Since you were diagnosed with diabetes, have you ever tried to quit alcohol? | 1 | Yes | |
|  |  | 2 | No **🡪 Proceed to question 48** | |
| **47** | How difficult was it for you?  ***(Read the options)*** | 1 | Very easy | |
|  |  | 2 | Easy | |
|  |  | 3 | Difficult | |
|  |  | 4 | Very difficult | |
| **48** | Since you were diagnosed with diabetes, have you ever tried to consume less fat or fried foods? | 1 | Yes | |
|  |  | 2 | No **🡪 Proceed to question 50** | |
| **49** | How difficult was it for you?  ***(Read the options)*** | 1 | Very easy | |
|  |  | 2 | Easy | |
|  |  | 3 | Difficult | |
|  |  | 4 | Very difficult | |
| **50** | Since you were diagnosed with diabetes, have you ever tried to consume more vegetables? | 1 | Yes | |
|  |  | 2 | No **🡪 Proceed to question 52** | |
| **51** | How difficult was it for you?  ***(Read the options)*** | 1 | Very easy | |
|  |  | 2 | Easy | |
|  |  | 3 | Difficult | |
|  |  | 4 | Very difficult | |
| **52** | Since you were diagnosed with diabetes, have you ever tried to lose weight? | 1 | Yes | |
|  |  | 2 | No **🡪 Proceed to question 55** | |
| **53** | What did you do to lose weight? |  | | |
| **54** | How easy or difficult was it to lose weight?  **(Read the options)** | 1 | Very easy | |
|  |  | 2 | Easy | |
|  |  | 3 | Difficult | |
|  |  | 4 | Very difficult | |
| **55** | Do you have a family member, friend or neighbour who helps you to control your diabetes? | 1 | Yes | |
|  |  | 2 | No**🡪 Proceed to question 58** | |
| **56** | Who | 1 | Spouse | |
|  |  | 2 | Partner | |
|  |  | 3 | Son | |
|  |  | 4 | Daughter | |
|  |  | 5 | Cousin | |
|  |  | 6 | Friend | |
|  |  | 7 | Neighbour | |
|  |  | 8 | Other | |
| **57** | How do they help you? |  | | |
| **58** | Imagine that you wanted to lose weight. If you had to choose a family member or friend to help you lose weight, who would you choose? | 1 | Spouse | |
|  |  | 2 | Partner | |
|  |  | 3 | Son | |
|  |  | 4 | Daughter | |
|  |  | 5 | Cousin | |
|  |  | 6 | Friend | |
|  |  | 7 | Neighbour | |
|  |  | 8 | Other | |
| **59** | Why would you choose that person? |  | | |

| **Section 4: Incentives** |
| --- |

| **Module: incentives** | | **Response** | |
| --- | --- | --- | --- |
| **60** | Imagine that we invite you to participate in a health programme in which we will give you a reward in soles if you achieve weight loss. This programme will last 9 months and the challenge is that you lose 1 kg every 2 weeks.  If I ask you to lose 1 kg every 2 weeks and I give you a cash reward when you achieve it… what amount of cash would motivate you to lose weight? | 1 | 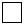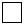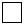soles |
|  |  | 2 | Doesn’t know/respond |

| **Question** | | **Response** | | |
| --- | --- | --- | --- | --- |
| **61** | And would you accept the challenge of losing 1 kg in 2 weeks if I paid you nothing? | | 1 | Yes |
|  |  |  | 2 | No |
| **62** | And would you accept the challenge of losing 1 kg in 2 weeks if I paid you 50 soles? | | 1 | Yes |
|  |  |  | 2 | No |
| **63** | And would you accept the challenge of losing 1 kg in 2 weeks if I paid you 100 soles? | | 1 | Yes |
|  |  |  | 2 | No |
| **64** | And would you accept the challenge of losing 1 kg in 2 weeks if I paid you 150 soles? | | 1 | Yes |
|  |  |  | 2 | No |
| **65** | And would you accept the challenge of losing 1 kg in 2 weeks if I paid you 200 soles? | | 1 | Yes |
|  |  |  | 2 | No |
| **66** | And would you accept the challenge of losing 1 kg in 2 weeks if I paid you 250 soles? | | 1 | Yes |
|  |  |  | 2 | No |
| **67** | Can you explain to me why you stopped at ___ soles? | | | |
| **68** | To participate it is necessary to be in the programme for 9 months. Would you be interested in participating in this weight loss programme for 9 months? Participation would cost you nothing. | 1 | Yes | |
|  |  | 2 | No | |
| **69** | What would be the best way of providing the reward  ***(Read the options, choose only one)*** | 1 | Cash | |
|  |  | 2 | Deposit in bank account | |
|  |  | 3 | Vouchers | |
|  |  | 4 | Other: | |
| **70** | Would you agree to share the reward with another person who helped you to lose weight> | 1 | Yes | |
|  |  | 2 | No | |
|  | If you had to share the reward with somebody who helped you to lose weight, how much would you give them>  ***(Read the options, choose only one)*** | 1 | More than half | |
|  |  | 2 | Half | |
|  |  | 3 | Less than half | |
|  |  | 4 | All | |
|  |  | 5 | Nothing | |

| **72** | Imagine that we invite you to participate in a health programme in which we give you a reward in soles if you achieve weight loss.  In this opportunity we will make a contract with you in which we ask you to deposit an amount in a savings account. If you achieve 1 kg weight loss in 2 weeks we will double your deposit. For example, if you have deposited 5 soles and lose 1 kg, we will give you 10 soles.  If you do not achieve the target, you will lose your deposit.  You could only participate if you agreed to participate for 9 months.  Would you be willing to participate? | 1 | Yes |
| --- | --- | --- | --- |
|  |  | 2 | No |
| **73** | If you participated in this programme, how much money would you be willing to deposit? | 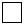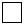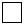 Soles | |

| **Pregunta** | | **Respuesta** | |
| --- | --- | --- | --- |
| **74** | Would you deposit 25 soles to win 50 soles if you lost 1 kg in 2 weeks? | 1 | Yes |
|  |  | 2 | No |
| **75** | Would you deposit 50 soles to win 100 soles if you lost 1 kg in 2 weeks? | 1 | Yes |
|  |  | 2 | No |
| **76** | Would you deposit 75 soles to win 150 soles if you lost 1 kg in 2 weeks? | 1 | Yes |
|  |  | 2 | No |
| **77** | Would you deposit 100 soles to win 200 soles if you lost 1 kg in 2 weeks? | 1 | Yes |
|  |  | 2 | No |
| **78** | Would you deposit 150 soles to win 300 soles if you lost 1 kg in 2 weeks? | 1 | Yes |
|  |  | 2 | No |
| **79** | Would you deposit 200 soles to win 250 soles if you lost 1 kg in 2 weeks? | 1 | Yes |
|  |  | 2 | No |
| **80** | Would you deposit 250 soles to win 500 soles if you lost 1 kg in 2 weeks? | 1 | Yes |
|  |  | 2 | No |
| **81** | Can you explain why you stopped at ___ soles? | | |

**Notes:**
