# Supplementary material for: A descriptive study of potential participant preferences for the design of an incentivised weight loss programme for people with type 2 diabetes mellitus attending a public hospital in Lima, Peru
Source: Wellcome Open Res. 2018 Sep 27;3:53. Originally published 2018 May 3. [Version 2] doi: 10.12688/wellcomeopenres.14552.2 (PMC6348435; doi:10.12688/wellcomeopenres.14552.2)
Supplement: Supplementary file 4 [file wellcomeopenres-3-16187-s0003.tgz › 49fc6df0-a4ed-45bc-b8d1-ac429289b3f8_Supplementary_File_2.doc]

**CRÓNICAS**

**CENTRO DE EXCELENCIA EN ENFERMEDADES CRÓNICAS**

**Estudio Formativo Parte 1**

**Incentivos monetarios para personas con Diabetes**

Por favor preséntese antes de empezar:

*“Buenos días / tardes, mi nombre es …. Soy personal de salud del Centro de Excelencia en Enfermedades Crónicas de la Universidad Peruana Cayetano Heredia. Estamos realizando un estudio de investigación sobre el uso de premios para promover que las personas con diabetes tengna un mejor control de su enfermedad.*

*Usted ha sido contactado porque tiene diabetes mellitus tipo 2 y porque recibe atención dentro de este establecimiento de salud.*

*Me gustaría hacerle unas preguntas sobre sus datos generales. Antes le explicaré las razones del estudio y le consultaré si está interesado en participar. Luego le daré una hoja informativa sobre el estudio y Ud. decidirá si desea participar.*

**Sección 1: Datos de la Aplicación y del Participante**

| **Módulo: Datos de la Aplicación** | | **Respuestas** | | | | | |
| --- | --- | --- | --- | --- | --- | --- | --- |
| **1** | Código de la entrevistadora |  |  | | | | |
| **2** | Fecha (DD-MMM-20AA) |  |  | **-** |  | **-** |  |

| **Módulo: Información demográfica** | | **Respuestas** | | | | | |
| --- | --- | --- | --- | --- | --- | --- | --- |
| **3** | Sexo (registre de acuerdo a lo observado) | 1 | Hombre |  |  |  |  |
|  |  | 2 | Mujer |  |  |  |  |
| **4** | Fecha de Nacimiento (DD-MMM-20AA) |  |  | **-** |  | **-** |  |
| **5** | Años cumplidos a la fecha… |  Años | | | | | |

| **Módulo: Consentimiento** | | **Respuestas** | |
| --- | --- | --- | --- |
| **6** | Se ha leído el consentimiento | 1 | Si |
|  |  | 2 | No **🡪 Leer consentimiento** |
| **7** | Se ha obtenido el consentimiento del participante | 1 | Si |
|  |  | 2 | No **🡪 Terminar la entrevista** |

***La información contenida en esta sección debe guardarse separada del cuestionario ya que contiene información confidencial.***

| **Sección 2: Datos Demográficos y Socioeconómicos** | | | |
| --- | --- | --- | --- |
| **Módulo: Información demográfica** | | **Respuesta** | |
| **8** | ¿Cuál es su estado civil? | 1 | Soltero |
|  |  | 2 | Casado |
|  |  | 3 | Conviviente |
|  |  | 4 | Separado |
|  |  | 5 | Divorciado |
|  |  | 6 | Viudo |
|  |  | 99 | Rehúsa responder |
| **9** | ¿Con qué seguro de salud cuenta usted?  ***(Lea las opciones)*** | 1 | SIS |
|  |  | 2 | Essalud |
|  |  | 3 | Ninguno |
|  |  | 4 | Otro: |
| **10** | ¿Cuál es el nivel de educación más alto que ha alcanzado?  ***(Marcar solo una opciòn)*** | 1 | Sin nivel |
|  |  | 2 | Educación inicial |
|  |  | 3 | Primaria incompleta |
|  |  | 4 | Primaria completa |
|  |  | 5 | Secundaria incompleta |
|  |  | 6 | Secundaria completa |
|  |  | 7 | Superior no universitaria incompleta |
|  |  | 8 | Superior no universitaria completa |
|  |  | 9 | Superior universitaria incompleta |
|  |  | 10 | Superior universitaria completa |
|  |  | 88 | No sabe / No recuerda |
|  |  | 99 | Rehúsa responder |
| **11** | ¿Tiene usted algún trabajo o empleo, fijo o eventual, por el que reciba un sueldo o ingreso? | 1 | Si |
|  |  | 2 | No |
| **12** | ¿Cuál es su ocupación o actividad principal?  ***(Marcar solo una opción)*** | 1 | Empleado del gobierno |
|  |  | 2 | Empleado del sector privado |
|  |  | 3 | Trabajador independiente |
|  |  | 4 | Obrero |
|  |  | 5 | Campesino |
|  |  | 6 | Ama de casa |
|  |  | 7 | Trabajador/a del hogar |
|  |  | 8 | Otros: __________________________ |
|  |  | 99 | Rehúsa responder |
| **13** | Además de su sueldo, ¿Usted, tiene alguna otra fuente de ingreso adicional?  ***(Lea las opciones, marque todas las que correspondan)*** | 1 | Alquiler de vivienda |
|  |  | 2 | Alquiler de equipos |
|  |  | 3 | Alquiler de tierras |
|  |  | 4 | Jubilación |
|  |  | 5 | Pensión 65 |
|  |  | 6 | Remesas de otros familiares |
|  |  | 7 | Pensión por alimentos, separación, viudez, discapacidad |
|  |  | 8 | Otros: |
|  |  | 9 | No tiene otros ingresos |
|  |  | 99 | Rehúsa responder |
| **14** | ¿Los ingresos que usted genera son para gastos personales, para gastos familiares o ambos? | 1 | Gastos personales |
|  |  | 2 | Gastos familiares |
|  |  | 3 | Ambos |
|  |  | 4 | No genera ingresos |
| **15** | Incluyéndose a usted, ¿cuántas personas viven en su hogar? No considere a las trabajadoras del hogar |  personas | |
| **16** | Incluyéndose a usted, ¿cuántas personas que viven en su hogar tienen ingresos económicos? |  personas tienen ingresos económicos | |
| **17** | Sume los ingresos mensuales **de todas las personas de su hogar**: Usted, su pareja, sus hijos, sus padres, etc. Incluya todas las fuentes de ingreso: sueldos, pensiones, apoyo económico de familiares, rentas, etc..  ¿Cuál es el ingreso mensual de su hogar?  ***(Lea las opciones. Marque solo una)*** | 1 | < = 750 soles |
|  |  | 2 | Entre 751 y 1500 soles |
|  |  | 3 | Entre 1501 y 2000 soles |
|  |  | 4 | Entre 2001 y 2500 soles |
|  |  | 5 | > 2501 soles |
|  |  | 88 | No sabe / No recuerda |
|  |  | 99 | Rehúsa responder |
| **18** | ¿Cómo diría Ud. que es la situación económica de su hogar en la actualidad?  ***(Lea las opciones. Marque solo una)*** | 1 | Muy buena |
|  |  | 2 | Buena |
|  |  | 3 | Regular |
|  |  | 4 | Mala |
|  |  | 5 | Muy mala |

**Sección 3: Salud**

| **Módulo: Salud** | | **Respuesta** | | |
| --- | --- | --- | --- | --- |
| **19** | ¿Usted diría que su SALUD, el día de HOY, es…?  ***(Lea las opciones. Marque sólo una)*** | 0 | Muy Mala | |
|  |  | 1 | Mala | |
|  |  | 2 | Regular | |
|  |  | 3 | Buena | |
|  |  | 4 | Muy Buena | |
|  |  | 98 | No sabe / No responde | |
| **20** | ¿Sabe Ud. cuál es su peso actual aproximadamente? | 1 | Si | |
|  |  | 2 | No **🡪 Pasar a la pregunta 22** | |
| **21** | ¿Aproximadamente, cuánto pesa usted? | **.** [Kg] | | |
| **22** | ¿A usted le gustaría…?  ***Lea las opciones. Marque sólo una)*** | 1 | Bajar de peso | |
|  |  | 2 | Mantener su peso actual | |
|  |  | 3 | Subir de peso | |
|  |  | 98 | No sabe / No responde | |
| **23** | ¿Sabe Ud. cuánto mide aproximadamente? | 1 | Si | |
|  |  | 2 | No **🡪 Pasar a la pregunta 25** | |
| **24** | ¿Aproximadamente, cuánto mide usted? |  [Cm] | | |
| **25** | ¿Hace cuánto tiempo le diagnosticaron diabetes mellitus? | Hace años o | | Hace  meses |
| **26** | En los últimos 7 días, ¿se ha medido (o le han medido) la glucosa? | 1 | Si | |
|  |  | 2 | No **🡪 Pasar a la pregunta 28** | |
|  |  | 98 | No sabe / No responde **🡪 Pasar a la pregunta 28** | |
| **27** | ¿Cuál fue su medida de glucosa? | (mg /dl) | | |
|  |  | 98 | No recuerda | |
| **28** | En los últimos 3 meses, ¿Usted se ha tomado la prueba de hemoglobina glicosilada? | 1 | Si | |
|  |  | 2 | No**🡪 Pasar a la pregunta 30** | |
|  |  | 98 | No sabe / No responde **🡪 Pasar a la pregunta 30** | |
| **29** | ¿Cuál fue su medida de hemoglobina glicosilada? |  .  % | | |
|  |  | 98 | No recuerda | |
| **30** | En estos momentos, ¿algún médico le ha indicado algún tratamiento para controlar su diabetes? | 1 | Si | |
|  |  | 2 | No **🡪 Pasar al siguiente módulo** | |
| **31** | ¿Está tomando o siendo tratado con alguno de los siguientes medicamentos?  **(Leer las opciones. Marque todas las que correspondan más de una opción)** | 1 | Metformina (pastilla) | |
|  |  | 2 | Glibenclamida (pastilla) | |
|  |  | 3 | Insulina | |
|  |  | 4 | Otros medicamentos: | |
|  |  | 5 | Pastillas para bajar de peso | |
|  |  | 98 | No está tomando medicamentos | |
| **32** | ¿Usted está tomando o usando estos medicamentos tal y como le indicó su médico? | 1 | Si | |
|  |  | 2 | No | |
|  |  | 3 | A veces | |
| **33** | ¿En promedio, cuánto gasta usted al mes para comprar sus medicamentos para diabetes? | Soles | | |
| **Módulo: Conocimientos sobre Diabetes** | | **Respuesta** | | |
| **35** | ¿Me puede decir tres formas en las que un paciente con diabetes puede controlar su enfermedad o mejorar su salud? | A |  | |
|  |  | B |  | |
|  |  | C |  | |
| **36** | ¿Usted practica algunas de las acciones que me nombró? | 1 | Si | |
|  |  | 2 | No**🡪 Pasar a la pregunta 38** | |
| **37** | ¿Cuál o cuáles practica usted?  **(Marque todas las que correspondan más de una opción)** | 1 | A | |
|  |  | 2 | B | |
|  |  | 3 | C | |
|  |  | 4 | Todas | |
| **38** | ¿Usted alguna vez ha recibido educación en temas de diabetes? | 1 | Si | |
|  |  | 2 | No **🡪 Pasar a la pregunta 42** | |
| **39** | ¿Dónde? |  | | |
| **40** | ¿Cuánto tiempo duró? (llenar sólo una opción) |  días | | |
|  |  | semanas | | |
|  |  | meses | | |
|  |  |  anos | | |
| **41** | ¿Quién le enseñó? |  | | |
| **42** | ¿Desde que le diagnosticaron diabetes, ha intentado alguna vez hacer ejercicios o actividad física con regularidad? | 1 | Si | |
|  |  | 2 | No **🡪 Pasar a la pregunta 44** | |
| **43** | ¿Qué tan fácil o difícil fue para usted?  ***(Lea las opciones)*** | 1 | Muy fácil | |
|  |  | 2 | Fácil | |
|  |  | 3 | Difícil | |
|  |  | 4 | Muy difícil | |
| **44** | Desde que le diagnosticaron diabetes ¿ha intentado alguna vez consumir menos azúcar o dulces? | 1 | Si | |
|  |  | 2 | No **🡪 Pasar a la pregunta 46** | |
| **45** | ¿Qué tan fácil o difícil fue para usted?  ***(Lea las opciones)*** | 1 | Muy fácil | |
|  |  | 2 | Fácil | |
|  |  | 3 | Difícil | |
|  |  | 4 | Muy difícil | |
| **46** | Desde que le diagnosticaron diabetes ¿ha intentado alguna vez dejar de consumir alcohol? | 1 | Si | |
|  |  | 2 | No **🡪 Pasar a la pregunta 48** | |
| **47** | ¿Qué tan fácil o difícil fue para usted?  ***(Lea las opciones)*** | 1 | Muy fácil | |
|  |  | 2 | Fácil | |
|  |  | 3 | Difícil | |
|  |  | 4 | Muy difícil | |
| **48** | Desde que le diagnosticaron diabetes, ¿Ha intentado alguna vez consumir menos grasas o frituras? | 1 | Si | |
|  |  | 2 | No **🡪 Pasar a la pregunta 50** | |
| **49** | ¿Qué tan fácil o difícil fue para usted?  ***(Lea las opciones)*** | 1 | Muy fácil | |
|  |  | 2 | Fácil | |
|  |  | 3 | Difícil | |
|  |  | 4 | Muy difícil | |
| **50** | Desde que le diagnosticaron diabetes ¿Ha intentado alguna vez consumir más verduras? | 1 | Si | |
|  |  | 2 | No **🡪 Pasar a la pregunta 52** | |
| **51** | ¿Qué tan fácil o difícil fue para usted?  ***(Lea las opciones)*** | 1 | Muy fácil | |
|  |  | 2 | Fácil | |
|  |  | 3 | Difícil | |
|  |  | 4 | Muy difícil | |
| **52** | Desde que le diagnosticaron diabetes ¿Ha intentado alguna vez bajar de peso? | 1 | Si | |
|  |  | 2 | No **🡪 Pasar a la pregunta 55** | |
| **53** | ¿Qué hizo para bajar de peso? |  | | |
| **54** | ¿Qué tan fácil o difícil fue para usted tratar de bajar de peso?  **(Lea las opciones)** | 1 | Muy fácil | |
|  |  | 2 | Fácil | |
|  |  | 3 | Difícil | |
|  |  | 4 | Muy difícil | |
| **55** | ¿Tiene usted algún familiar, amigo o vecino que le ayuda a controlar su diabetes? | 1 | Si | |
|  |  | 2 | No**🡪 Pasar a la pregunta 58** | |
| **56** | ¿Quién? | 1 | Esposo/Esposa | |
|  |  | 2 | Pareja | |
|  |  | 3 | Hijo | |
|  |  | 4 | Hija | |
|  |  | 5 | Sobrino/Sobrina | |
|  |  | 6 | Amigo/ amiga | |
|  |  | 7 | Vecino/vecina | |
|  |  | 8 | Otro: | |
| **57** | ¿Cómo lo ayudan? |  | | |
| **58** | Imagínese que usted quiere bajar de peso. Si tuviera que elegir a alguien de su familia o a algún amigo(a) para que lo ayude a bajar de peso. ¿A quién elegiría? | 1 | Esposo/Esposa | |
|  |  | 2 | Pareja | |
|  |  | 3 | Hijo | |
|  |  | 4 | Hija | |
|  |  | 5 | Sobrino/Sobrina | |
|  |  | 6 | Amigo/ amiga | |
|  |  | 7 | Vecino/vecina | |
|  |  | 8 | Otro: | |
| **59** | ¿Por qué elegiría a esa persona? |  | | |

**Sección 4: Incentivos**

| **Módulo: Incentivos** | | **Respuesta** | |
| --- | --- | --- | --- |
| **60** | Imagínese que lo invitamos a inscribirse en un programa de salud en el que le daremos un premio en soles si consigue bajar de peso. Este programa durará 9 meses y la meta es que usted baje 1 kilo cada dos semanas.  Si le pido que baje 1 kilo cada dos semanas y le doy un premio en efectivo cuando lo logre… ¿Qué monto de dinero le animaría a bajar de peso? | 1 | soles |
|  |  | 2 | No sabe/ No responde |

| **Pregunta** | | **Respuesta** | | |
| --- | --- | --- | --- | --- |
| **61** | ¿Y aceptaría el reto de bajar 1 kilo en dos semanas si no le pago nada? | | 1 | Si |
|  |  |  | 2 | No |
| **62** | ¿Y aceptaría el reto de bajar 1 kilo en dos semanas si le pago 50 soles? | | 1 | Si |
|  |  |  | 2 | No |
| **63** | ¿Y aceptaría el reto de bajar 1 kilo en dos semanas si le pago 100 soles? | | 1 | Si |
|  |  |  | 2 | No |
| **64** | ¿Y aceptaría el reto de bajar 1 kilo en dos semanas si le pago 150 soles? | | 1 | Si |
|  |  |  | 2 | No |
| **65** | ¿Y aceptaría el reto de bajar 1 kilo en dos semanas si le pago 200 soles? | | 1 | Si |
|  |  |  | 2 | No |
| **66** | ¿Y aceptaría el reto de bajar 1 kilo en dos semanas si le pago 250 soles? | | 1 | Si |
|  |  |  | 2 | No |
| **67** | ¿Me puede explicar por qué paró en ______ soles? | | | |
| **68** | Para participar es necesario estar en el programa por 9 meses. ¿Usted estaría interesado en participar en este programa para bajar de peso por 9 meses? Debe saber que participar no tendrá ningún costo para usted**.** | 1 | Si | |
|  |  | 2 | No | |
| **69** | ¿Cuál sería la mejor manera de entregarle el premio?  ***(Lea las opciones, marque solo una)*** | 1 | En efectivo | |
|  |  | 2 | Depósito en una cuenta bancaria | |
|  |  | 3 | En un vale de compras | |
|  |  | 4 | Otros: | |
| **70** | ¿Estaría de acuerdo con compartir el premio con otra persona que lo ayude a bajar de peso? | 1 | Si | |
|  |  | 2 | No | |
| **71** | Si tuviera que compartir el premio con alguien que lo ayude a bajar de peso. ¿Cuánto le daría a esta persona?  ***(Lea las opciones, marque solo una)*** | 1 | Más de la mitad del premio | |
|  |  | 2 | La mitad del premio | |
|  |  | 3 | Menos de la mitad del premio | |
|  |  | 4 | Todo | |
|  |  | 5 | Nada | |

| **72** | Imagínese que lo invitamos a inscribirse en un programa de salud en el que le daremos un premio en soles si consigue bajar de peso.  En esta oportunidad haríamos un contrato y le pediríamos que usted deposite un monto en una cuenta de ahorros en el banco. Si usted logra bajar 1 kilo en dos semanas nosotros le duplicaremos el monto que usted ha depositado. Por ejemplo, si usted ha depositado 5 soles y baja 1 kilo, nosotros le damos 10 soles.  Si usted no llega a la meta, perdería el dinero que ha depositado.  Usted sólo podría participar si acepta participar a lo largo de 9 meses.  ¿Estaría dispuesto a participar? | 1 | Si |
| --- | --- | --- | --- |
|  |  | 2 | No |
| **73** | Si usted participara en este programa ¿Cuánto dinero estaría dispuesto a depositar en la cuenta de ahorros? |  Soles | |

| **Pregunta** | | **Respuesta** | |
| --- | --- | --- | --- |
| **74** | ¿Pondría 25 soles de su bolsillo para ganar 50 soles si baja un kilo en dos semanas? | 1 | Si |
|  |  | 2 | No |
| **75** | ¿Pondría 50 soles de su bolsillo para ganar 100 soles si baja un kilo en dos semanas? | 1 | Si |
|  |  | 2 | No |
| **76** | ¿Pondría 75 soles de su bolsillo para ganar 150 soles si baja un kilo en dos semanas? | 1 | Si |
|  |  | 2 | No |
| **77** | ¿Pondría 100 soles de su bolsillo para ganar 200 soles si baja un kilo en dos semanas? | 1 | Si |
|  |  | 2 | No |
| **78** | ¿Pondría 150 soles de su bolsillo para ganar 300 soles si baja un kilo en dos semanas? | 1 | Si |
|  |  | 2 | No |
| **79** | ¿Pondría 200 soles de su bolsillo para ganar 400 soles si baja un kilo en dos semanas? | 1 | Si |
|  |  | 2 | No |
| **80** | ¿Pondría 250 soles de su bolsillo para ganar 500 soles si baja un kilo en dos semanas? | 1 | Si |
|  |  | 2 | No |
| **81** | ¿Me puede explicar por qué paró en ____ soles? | | |

**Observaciones: (no digitar, lo digitará la encuestadora y lo enviará en un archivo aparte).**
