# Supplementary material for: A descriptive study of potential participant preferences for the design of an incentivised weight loss programme for people with type 2 diabetes mellitus attending a public hospital in Lima, Peru
Source: Wellcome Open Res. 2018 Sep 27;3:53. Originally published 2018 May 3. [Version 2] doi: 10.12688/wellcomeopenres.14552.2 (PMC6348435; doi:10.12688/wellcomeopenres.14552.2)
Supplement: Peer review history

# Peer review: Referee response for version 2

**DOI:** 10.21956/wellcomeopenres.16187.r33985

**Published:** 2018-10-16

**Authors:** Luzia Valentini, Sara Ramminger

**Affiliations:** 1 Institute of Evidence-based Dietetics, University of Applied Sciences Neubrandenburg, Neubrandenburg, Germany; 2 University of Applied Sciences Neubrandenburg, Neubrandenburg, Germany

The authors implemented most of the relevant comments, but still do not offer any hint in regard to the non linearity of weight loss and the monetary advantage of people with higher BMI in the applied incentivized program. The authors claim that that they have alluded to it in the revised version but we were not able to identify anything on it. Also some suggestions on who is going to provide the funding for monetary incentives in future is still missing and would be beneficial to add to the discussion part.

We have read this submission. We believe that we have an appropriate level of expertise to confirm that it is of an acceptable scientific standard.

# Peer review: Referee response for version 2

**DOI:** 10.21956/wellcomeopenres.16187.r33986

**Published:** 2018-10-15

**Authors:** Ramfis Nieto-Martínez

**Affiliations:** 1 South Florida Veterans Affairs Foundation for Research & Education (SFVAFRE) and Geriatric Research, Education, and Clinical Center (GRECC), Miami VA Healthcare System, Miami, FL, USA; 2 Department of Global Health and Population, Harvard TH Chan School of Public Health, Harvard University, Boston, MA, USA; 3 Department of Physiology, School of Medicine, University Centro-Occidental “Lisandro Alvarado” and Cardio-metabolic Unit 7, Barquisimeto, Venezuela

Thanks for considering the suggestions. The author changes are sufficient to amend the status of Approved with Reservations that I previously.

I have read this submission. I believe that I have an appropriate level of expertise to confirm that it is of an acceptable scientific standard.

# Peer review: Referee response for version 1

**DOI:** 10.21956/wellcomeopenres.15839.r33690

**Published:** 2018-08-15

**Authors:** Ramfis Nieto-Martínez

**Affiliations:** 1 South Florida Veterans Affairs Foundation for Research & Education (SFVAFRE) and Geriatric Research, Education, and Clinical Center (GRECC), Miami VA Healthcare System, Miami, FL, USA; 2 Department of Global Health and Population, Harvard TH Chan School of Public Health, Harvard University, Boston, MA, USA; 3 Department of Physiology, School of Medicine, University Centro-Occidental “Lisandro Alvarado” and Cardio-metabolic Unit 7, Barquisimeto, Venezuela

This article explores the acceptability and preferences of a group of 100 patients with type 2 diabetes who attended the endocrinology service in a public hospital in Peru, regarding a hypothetical monetary-incentivized weight loss program to be applied in the future. One of the gaps that, in the view of the authors, warrant obtaining this information is the necessity of strategies that increase the adherence to and sustainability of a weight loss intervention. Considering the innovation of the information based on an original questionnaire this article deserves publication. However, several important observations need to be considered:

**INTRODUCTION** 

1. **Number and quality of references**
  The authors should avoid loading the introduction of references. The introduction 22 references. All of them are necessary? 4 to 5 good quality (primary sources, review or meta-analyses) references is enough. For example:
  a) **Page 3, left column, paragraph 1, line 2**: "Weight control is critical for both the prevention and treatment of type 2 diabetes mellitus (T2DM) (1-4)."
  There are 4 references supporting it. The first reference is a report. The second is appropriate. References 3 and 4 are secondary sources belonging to sections of the American Diabetes Association Guidelines. I suggest looking for a systematic review supporting the importance of weight control in the prevention and treatment of T2D. 
  b) **Page 3, left column, paragraph 1, line 2-5**." Self-management programs for people with T2DM commonly include the promotion of lifestyle changes, such as dietary modifications and increasing physical activity, to reduce weight (5-7)."
  There are 3 references but none of them is a primary source. The reference 5 is ADA guidelines and the other 2 are web-pages containing information for patients. I suggest retiring these references (5,6,7) and include primary sources of studies demonstrating the effectiveness of structured weight loss interventions (not only self-management programs) on diabetes management. The most important structured interventions evaluating the effect of lifestyle changes on T2D management are the Look AHEAD study using HbA1c as main outcome and the Why Wait Study whose primary outcome was the weight ^1^^,^^2^. 
  c) **Page 3, left column, paragraph 1, line 5-6**. "However, sustained weight loss is a challenge to both patients and providers (8,9)."
  References 8 and 9 do not mention directly a sustained weight loss as a challenge. Both studies are interviews with physicians. In the reference 8, 14 GP were interviewed in focus groups. Five dilemmas were identified for primary physicians, but none of the 5 referred to difficulties of their patients in losing or maintaining weight loss. Reference 9 was an interview to 19 physicians to understand their challenges when treating social and emotional difficulties in T2D patients, but not the challenge of sustained weight loss to both patients and providers.
  It is very laborious as a reviewer to check all references one by one. I strongly recommend that all references be carefully reviewed to establish if they are relevant, useful and what is more important if they support the written statements.
2. **Background**
  In the introduction, the authors have to create a strong background that explains the reasoning behind why the study goal was built. In the introduction, the authors broadly mention sustainability as one of the possible benefits of adding incentives to a weight loss program. However, a probably greater sustainability of the loss of weight generated by the addition of incentives should not be proposed without bases that sustain it.
**Page 3, left column, paragraph 2, line 7-9. "**Sustained changes have been achieved through in the field of smoking cessation, although this remains a challenge to weight loss interventions."
  I consider that the comment regarding that sustainability in weight loss interventions is a challenge is very conservative. Two of the references cited deserves attention. In the reference 15, financial incentives in 66 US veterans with BMI between 30 and 40 and age between 30 and 70 years produced significant weight loss over an 8-month intervention; however, participants regained weight post-intervention. The reference 16 is a systematic review included nine randomized controlled trials of behavioral treatments for obesity and overweight involving the use of financial incentives with reported follow‐up of at least 1 year. No study was analyzed on an intention to treat basis, participants were mostly women recruited through media advertisements, mean age ranged from 35.7 to 52.8 years, and mean body mass index from 29.3 to 31.8 kg/m ^−2^. Results from meta‐analysis showed no significant effect of the use of financial incentives on weight loss or maintenance at 12 months and 18 months.
  With this background (under-registered in the article), the first important question in the introduction would be: In previous studies, monetary incentives have shown some benefit in patients who are in a weight loss program? If the answer is NO. Is it justified to evaluate it in this population since it has very different characteristics than those evaluated previously, and the results could be different? The same participants question the usefulness of the incentives to increase the willingness to participate since 92% of them reported agreeing to participate in a weight loss program without incentives. Also, 19% considered that. This aspect should be mentioned in the discussion.
3. **The gap of knowledge** 
  In the introduction, the authors must highlight the existing knowledge gap that this study will fill and the relevance of the research question. What is the gap? The need a questionnaire (or the generated information) to determine the acceptance of incentives or to explore the opinion of potential patients regarding the incentives? This gap must be the prelude the research question and the aim.
4. **The aim** 
  It should be brief and should make clear what is the question that your study tries to respond.
  The need for a questionnaire to determine the acceptability of monetary incentives and their characteristics within a weight loss plan? Although the introduction was designed to create the need for information on the design of a monetary incentive program for diabetic patients who would join a weight loss program, the first objective mentioned was the characterization of the population. I believe that they should be more direct and go to the main objective. Reconsider: we performed a questionnaire study of potential participants with the aim of defining their demographic, social, cultural and health characteristics…

 

**METHODS**

 a) I suggest including a sub-heading to explain the structure of the questionnaire and a summary of how was developed. 

 b) Inclusion criteria should be T2D diagnosed by laboratory tests available in the files of the endocrinology clinic and / or the use of antidiabetic drugs, and not only by self-report of the participant 

 c) Considering that the program is aimed at overweight and obese patients, why you did not consider a BMI <25 as an exclusion criterion? The perspective of the problem is different in a person of normal weight with respect to an overweight / obese person. How many of the included participants have a normal weight? 

**RESULTS**

 a) The results describe the demographics and socioeconomic characteristics of the T2D patients commonly seen in the endocrinology clinic, considering that was a convenience sample 

 b) Table 2 should include BMI, most recent glucose and HbA1c measurement taken from the hospital files instead of self-reported data 

 c) Table 3 shows that 53 participants reported regular exercise as a health control measure attempted, but when the frequency of difficulty of attempt to regular exercise is reported, the total of participants is 34. I observed the same discrepancy, in the reduction of sugar intake (75/65), to quit alcohol (31/29), to reduce fat intake (77/65). In the case of the increase of vegetable intake (57/57) and weight loss (42/42) no discrepancies between the number of participants reporting the behavior and the total of the three categories of difficulty. Please, correct it if it is a mistake. 

 d) In table 3 methods are included for weight loss control (3 for each participant). Why to avoid appointments and foot care are included? These methods have not a relationship with weight loss. 

**DISCUSSION**

 The discussion includes 1. How much the intervention (incentives) represent the proportion of personal disposable income (PDI)? 2. Explanation of the answers of participants about the methods of payment, 3. Comparison with other studies regarding the effect of a helper. I recommend:

 a) Re-organize the discussion

 b) Explain the economic utility theory more clearly

 c) Consider including in the discussion important topics below 

  

1. **Topic 1. Potential implementation**
  To provide some clues about the implementability, could you include a paragraph mentioning how is the usual care of the diabetic in the hospital and if the endocrinology clinic has a structured program of weight loss? How would monetary incentives be inserted into that program? Who could be the provider of the funds? If you plan to start as a research project?
  A Mayo Clinic group proposed a multispecialty outpatient Obesity Treatment Research Program for weight loss to be implemented in the next 5 years that initially will start with research funds ^3^.
  Future implementation of a monetary incentive strategy for weight loss must consider different elements. Identification of stakeholders and funding is essential. The Reach, Effectiveness, Adoption, Implementation, Maintenance (RE-AIM) Model for the evaluation of the effectiveness of interventions can be a proper framework to estimate the future impact of the incentive program. RE-AIM elements follow a sequence beginning with adoption and reach, followed by implementation and efficacy and finally maintenance. For this, it is important to establish the difference between the weight loss program and the incentive program. Please find attached a table summarising the possibilities of RE-AIM components being met by the incentives included in the weight-loss program.
2. **A model of behavioral changes to explain the effect of the intervention**
  Obesity is a chronic disease that, as hypertension or diabetes, must be treated for life. *The article does not mention the components of the future intervention*. Assuming that the intervention will be guided by current Obesity Clinical Practice Guidelines, lifestyle and behavioral changes (dietary and physical activity) and the use of obesity medications should be included. Behavioral changes are one of the cornerstones of the obesity management.
  The Stages of Change Model initially developed based on the experience of smokers who quit, propose that change in behavior occurs continuously through a process with several steps. Each step has a duration: pre-contemplation (6 mo), contemplation (6 mo), determination (1 mo), action (6 mo) and maintenance (6 mo). There are strategies that are more effective for each stage of change, and the goal is to reach the maintenance, the ideal stage of behavior. In the maintenance stage, people have sustained their behavior change (e.g. weight loss) for more than 6 months and intend to maintain the behavior and avoid relapsing. *Monetary incentives for 9 months as is proposed in the article could accelerate the initial steps but do not guarantee maintenance of behavioral changes that effectively let them maintain a healthy weight*. This is related to the comments about sustainability mentioned above. Please find attached a figure depicting the Stages of Change Model.
3. **Obesity as a complex disease**
  The present article is exploring the possible components of program but fail to mention the complexity of factors involved in the process of implementing this strategy to increase the adherence. The obesity per se is a complex disease with multiple pathways controlling individual feeding behavior. Also, there are physiological adaptations occurring after weight loss such as changes in body composition, hormonal environment, energy expenditure, and control of food intake that predispose to regain the weight loss. The only options showing sustainable results and low food availability and access that have occurred during crisis and famine periods, and the forced modification of gastrointestinal anatomy by bariatric surgery. To offer money to participants to stimulate their adherence need to be tested in the future compared with a group without incentives in a well-designed randomized clinical trial. It is possible that the strategy can be effective for a short time but difficult to maintain.

**CONCLUSION**

 The conclusion is very direct and only mentions that incentives seem to be a strategy widely accepted by the diabetic population of Lima. This is an excessive generalization considering the sampling and the observations I made in the last paragraph on the background.

 Remembering: Participants question the usefulness of the incentives to increase the willingness to participate since 92% of them reported agreeing to participate in a weight loss program without incentives. Also, 19% considered that is not good to receive money for taking care of the own health.

**LIMITATIONS**

 Based on my previous observations, review the limitations again. I leave it to your discretion to reconsider the need to include some additional aspect.

I have read this submission. I believe that I have an appropriate level of expertise to confirm that it is of an acceptable scientific standard, however I have significant reservations, as outlined above.

## References

[1] Look AHEAD Research GroupWingRR: Long-term effects of a lifestyle intervention on weight and cardiovascular risk factors in individuals with type 2 diabetes mellitus: four-year results of the Look AHEAD trial. *Arch Intern Med*.2010;170(17) : 10.1001/archinternmed.2010.334 1566-75 10.1001/archinternmed.2010.334 20876408PMC3084497

[2] HamdyOMottalibAMorsiAEl-SayedNGoebel-FabbriAArathuzikGShaharJKirpitchAZrebiecJ: Long-term effect of intensive lifestyle intervention on cardiovascular risk factors in patients with diabetes in real-world clinical practice: a 5-year longitudinal study. *BMJ Open Diabetes Res Care*.2017;5(1) : 10.1136/bmjdrc-2016-000259 e000259 10.1136/bmjdrc-2016-000259 28090332PMC5223646

[3] MikhailDSJensenTBWadeTWMyersJFFrankJMWielandMHensrudDMcMahonMMCollazo-ClavellMLAbu-LebdehHKennelKAHurleyDLGrotheKJensenMD: Methodology of a multispecialty outpatient Obesity Treatment Research Program. *Contemp Clin Trials Commun*.2018;10: 10.1016/j.conctc.2018.03.004 36-41 10.1016/j.conctc.2018.03.004 29696156PMC5898534

# Response

**Published:** 2018-09-22

**Authors:** Harold Akehurst

**Affiliations:** Great Western Hospitals NHS Trust, UK

Dear Dr Nieto-Martinez,

Many thanks for taking the time to offer your review of our paper. We have revised the manuscript and hope that we have addressed your concerns. We have revised the references in the introduction largely in accordance with your suggestions. We did not revise refs 5-7 (in the version you reviewed), which are intended to support the assertion that self-management programmes do incorporate lifestyle changes, rather than their efficacy, which is the subject of the preceding sentence. You express very understandable concern that anthropometric and laboratory data are not reported. We agree entirely that these are desirable. Our study did not have access to medical records, and to do so would have required significantly more manpower than could justifiably been expended for a small pilot study (the setting does not benefit from electronic medical records). The decision to restrict to a questionnaire was pragmatic. We have nonetheless elaborated on this limitation in the revised manuscript. Although the absence of independent verification of diabetes disease status does mean that theoretically respondents might not have actually been diabetic, we do consider this unlikely given their recruitment from a diabetes clinic, and that 95% were able to state that they were taking antidiabetic treatment. We are especially grateful for your scrutiny of Table 3, in which there were indeed a number of numerical errors which have been corrected. We have also corrected an error which you noticed in Table 3 about participants suggested methods for health control. You ask specifically about the response “Avoid appointments”, which I must confirm is correctly rendered. The logic is the participant’s own. You kindly offer three additional topics for discussion, some of which you will find included in the revised manuscript. Your suggestions about the logistics of weight loss interventions, the RE-AIM model, and the holistic management of obesity are clearly critical to the design and evaluation of an intervention such as that planned by our research group. Nevertheless, it is difficult to integrate these issues into a discussion of the data which our questionnaire study has generated. The discussion section is already as long as the introduction, methods and results combined, and out of concern not to ‘bury the lede’ we are reluctant to expand it beyond the scope of our exploration of participant preferences. Again, we are very grateful for your kind review.

Yours sincerely, Harold Akehurst

# Peer review: Referee response for version 1

**DOI:** 10.21956/wellcomeopenres.15839.r33312

**Published:** 2018-07-02

**Authors:** Luzia Valentini, Sara Ramminger

**Affiliations:** 1 Institute of Evidence-based Dietetics, University of Applied Sciences Neubrandenburg, Neubrandenburg, Germany; 2 University of Applied Sciences Neubrandenburg, Neubrandenburg, Germany

The present report deals with a patient survey on potential monetary incentives to increase adherence in future weight loss programs to address type 2 diabetic in poorer section of the population in Peru. To address the topic is thoughtful as well as important and it clearly deserves publication.

 Nevertheless, the survey itself leads to no clear solution. On one hand it shows clear results on the missing attractiveness of the deposit-contract scheme and that cash/bank transfer is preferred over vouchers. But on the other hand it offers also important insights into the prejudices towards monetary incentives as well as it potential benefits. Interestingly, the participants themselves addressed the potential short-lived nature of monetary incentives during the weight loss without addressing incentives to maintain weight afterwards. These results, therefore, are ambiguous and not disputed in the discussion section. The discussion and conclusion parts do not sufficiently address major points of the result section.  In hindsight the authors might have designed the questionnaire differently, offering more options, possibly also ones that address intrinsic motivation and not only extrinsic motivation, as shown in this report.

**Major points:**

**Ad 2) Study design**:

- Anthropometric data of the participants are missing. What was the mean BMI (standard deviation)? As BMI was not an inclusion criterion: How many respondents were in the normal weight, overweight, obese I, II and III range?
- There was a bias in the selection of participants, which was addressed in the discussion section. Apparently those who intended to lose weight agreed more often to participate in the survey as seen by the 94 %age who agreed to the unincentivised weight loss program and the skewed, high percentage of participating women. The selection bias might be unavoidable but means, that the researchers addressed “the converted” in the survey and not those who do not want to lose weight but, nevertheless, might motivated by the monetary payment. It might also mean, that motivation to participate in a weight loss program might not be achieved by alone by “money for kilos”. We miss the dispute on this issue in the discussion part. It there a way to reach the so far unreachable group and can it be achieved by monetary incentives at all?
- Weight loss is not linear but more at the beginning and less later on. The main weight loss is expected to happen in the first 3 months. Therefore, the rationale for the bi-weekly payment for 1 kg weight loss is not self-explanatory. It might demotivate at the beginning (when more than 1 kg is lost in the 2-week period) and at the end of the program (when weight loss slows down). Participants with higher BMI and more kg of potential meaningful weight loss can “earn” more money than participants in the overweight range, in whom a weight loss of 5-10 kg is adequate and this should be achieved in max. 20 weeks = 5 months   according to the suggesting scheme (which is in line with international guidelines). Meaning that it is achieved prior to the program end. Is this motivating? We miss the discussion about these issues in the discussion part.
- Results mention that 85% would participate in an incentivized weight loss program, but even more (92%) responded that they would participate in an UNincentivised weight loss program anyway (page 3, right column, last two lines). This result should be mentioned in the abstract (and discussed in the discussion part).
- Who is going to provide the monetary incentive? Is it realistic that the money will be available in long-term? Also this should be mentioned somewhere in the manuscript.

 

**Ad 3) Methods – details:**

- The main questionnaire is only available in the Spanish language – translation to the English language would be helpful
- The structure of the method section should be improved, for example there should be an extra bullet point for the questionnaire development
- The explanations about the questionnaire are sometimes confusing, maybe an overview/figure about the questioning techniques (direct and fixed increment) and the related issues could be helpful.
- Who conducted the interviews or were parts of the questionnaire completed by the participants themselves?
- Inclusions criteria: Why self-reported T2DM? The recruitment took place in the endocrinology department, so blood values could have been recorded (e.g. HbA1c)
- The body height and body weight was not documented and not asked?

 

**Ad 6) discussion**

 The discussion does not address all major results,. E.g. Results mention that 85% would participate in an incentivized weight loss program, but even more (92%) responded that they would participate in an unincentivised weight loss program (page 3, right column, last two lines). 14 participants (19%) further considered that it was not good to receive money for taking care of their own health and one explained that it was like “selling ourself” (page 7, left col, para 2, L3-109). Especially for women, being poor and “selling yourself” implies critical and serious connotations. Surprisingly, only 16 participants thought that payment was a good idea.  We think these are rather unexpected and important results, which were not addressed in the discussion part. Furthermore, five participants addressed that payment will achieve only short-term results and this was also not mention in the discussion section. It is opening an important discussion if “payment per kilo” or incremental payment DURING weight loss is indeed a promising solution or if other models might be more promising (payment AFTER achieving weight loss goals or payment (or reduction /or extra money for health care costs) during the maintenance period and on long-term. We miss discussions and critical reflections on these issues in the discussion part.

 Also the conclusion is irritating and does not reflect the results.

**Minor comments:**

Abstract:

- The exact formulation of objectives is missing.
- Result on the 92% that responded that they would participate in an unincentivised weight loss program (page 3, right column, last two lines) should be added.
- Add “each” between “incentive for” and “1 kg”, otherwise it is unclear if the incentives incremental and paid every 2 weeks and not as one payment after 9 months.
- Conclusion is irritating and does not reflect results (see also above ad 6.)

Introduction:

- First paragraph focusses mainly on sustainability of weight loss, which is not addressed by the survey.
- At the beginning or rather in the rationale, it is initially not clear that the questionnaire deals with financial incentives
- You quoted an interesting systematic review about the financial incentive in treatment of obesity and overweight (John KL, et al., J Gen Intern Med 2011). Which results or conclusions were reached by the review?
- You say social and cultural factors influence the participant’s engagement with weight control, what exactly are the consequences?
- It would also be interesting to know how the T2DM patients are normally cared for in Peru in addition to medical treatment. Are there any dietetic interventions by dietitians? Are there accompanying weight loss programs common? What does basic health insurance cover and what have patients normally pay by themselves?
- Reading the title and the manuscript as a whole, the main aim as to our understanding was to investigate the attractiveness of monetary incentives in weight loss programs, whereas defining the demographic, social, cultural and health characteristics was secondary and only accessory (and therefore rather rough). Therefore, the phrasing of the aims is irritating to us.
- It is unclear if the incentivized weight loss program is intended for research purposes first and for integration in a regular health care program later on OR is it intended for research purposes only OR is it intended to start straight with a regular health care program? Information on this would be helpful.

Results:

- As already mentioned in major comment 1) we miss the BMI/body weight data of the participants.
- Page 4, left col, L4-5: wording “because they thought 9 months was a long time to avoid craving” is unclear
- Table 2: Why weren't the laboratory values taken from the hospital patient records?
- Table 3: it would have been also interesting to know the reason WHY the participants found it difficult to implement the health control measures, if they tried and even if they have not yet tried. We miss this point in the limitation section of the discussion. We also wondered about some answers on the methods for weight loss control. Why are “avoid appointment” and “foot care” listed? A short explanation would be helpful

 

Discussion:

- See also major point 6. Major issues are not discussed and the conclusion is irritating.
- Page 7, left col, last para: What is meant by “the second method”?

The discussion remains vague, for example it is nice to read about the different theories about behavior change, but where is the link to the results or the conclusion?

We have read this submission. We believe that we have an appropriate level of expertise to confirm that it is of an acceptable scientific standard, however we have significant reservations, as outlined above.

# Response

**Published:** 2018-09-22

**Authors:** Harold Akehurst

**Affiliations:** Great Western Hospitals NHS Trust, UK

Dear Professor Valentini,

Thank you very much for taking the time to offer your helpful report on our paper. We have revised the manuscript and hope that we have addressed your concerns.

You raise a very interesting point about how identical payments at equal intervals with the same target for all participants do not correspond to the non-linearity of weight loss over time, or to the differential weight loss requirements of people with different BMI. I have alluded to this in this revision but held back from a more detailed discussion which I think would exceed the scope of our results, which do not provide much material on which to base such a discussion.

Yours sincerely,

Harold Akehurst
